# Ectoine-Containing Inhalation Solution versus Saline Inhalation Solution in the Treatment of Acute Bronchitis and Acute Respiratory Infections: A Prospective, Controlled, Observational Study

**DOI:** 10.1155/2019/7945091

**Published:** 2019-01-31

**Authors:** Binh-Hai Tran, Van-Anh Dao, Andreas Bilstein, Klaus Unfried, Kija Shah-Hosseini, Ralph Mösges

**Affiliations:** ^1^Institute of Medical Statistics and Computational Biology, Faculty of Medicine, University of Cologne, Kerpener Straße 62, 50937 Cologne, Germany; ^2^CRI – Clinical Research International Ltd., Genter Str. 7, 50672 Cologne, Germany; ^3^Bitop AG, Carlo-Schmid-Allee 5, 44263 Dortmund, Germany; ^4^IUF Leibniz Research Institute for Environmental Medicine, Auf'm Hennekamp 50, 40225 Düsseldorf, Germany

## Abstract

**Purpose:**

This study investigated an inhalation solution containing ectoine, a bacterial-derived extremolyte, for the treatment of acute bronchitis and acute respiratory infections in comparison with saline inhalation solution.

**Methods:**

This prospective, controlled, observational study comprised an inclusion visit (day 1), a final visit (day 7), and a follow-up questionnaire (day 17). The treatment itself was administered from day 1 to day 7. The Bronchitis Severity Score, patients' general health, general effectiveness of the treatment, tolerability, and adverse events were compared between two groups.

**Results:**

In total, 135 patients were recruited; 79 patients received ectoine inhalation solution and 56 saline inhalation solution. After treatment, symptom scores decreased significantly in both groups (*P* < 0.05); the reduction in symptom scores was slightly greater in the ectoine group than in the saline group. The first significant reduction in symptom scores (*P* < 0.05) occurred earlier in the ectoine group than in the saline group. The differences in the area under the curve for the symptoms of dyspnea and auscultation findings were significant in favor of ectoine (*P* < 0.05). After treatment, more patients and physicians in the ectoine group assessed their or their patients' condition as “completely recovered” or “greatly improved” than those in the saline group. Almost all patients and physicians assessed the tolerability of both treatments as “good” or “very good”.

**Conclusions:**

Ectoine inhalation solution seems to be slightly more effective than saline inhalation solution for the treatment of acute bronchitis and acute respiratory infections.

## 1. Introduction

Acute bronchitis and acute respiratory infections are among the most common diseases for which patients seek medical advice. Respiratory infections cause 30% to 40% of all doctor visits in the UK and 75–100 million doctor visits per year in the US [1]. Acute bronchitis and acute respiratory infections are self-limited diseases typically persisting for about 2 weeks. Thus, treatment for acute bronchitis mainly comprises symptom control, improving patients' quality of life, and preventing further inflammations.

Ectoine is a natural extremolyte with inflammation-reducing properties. It was first found in microorganisms from extreme osmotic habitats. Ectoine binds strongly to water molecules and forms a hydrate shield that prevents dehydration and proteins' irreversible denaturation [2, 3]. Studies in bacteria have shown that ectoine is produced in response to high salt concentrations and extreme temperatures [4, 5]. Studies in cell culture and rodents have also shown that ectoine protects lung and skin cells against the invasion of nanoparticles and allergens [6–9]. Ectoine has versatile applications in medicine: it is used in cream for dermatitis and antiaging [10, 11], in nasal spray for acute rhinosinusitis [12], and in nasal spray and eye drops for allergic rhinitis [13]. Furthermore, it has been envisaged as a therapy in neurodegenerative disorders [14] and organ transplantation [15].

The ectoine inhalation solution (Ectoin® inhalation solution, bitop AG, Dortmund, Germany) used in this study is a registered medical device indicated for the treatment of airway inflammations. In patients with neutrophilic lung inflammations, the application of ectoine inhalation solution significantly reduced the levels of two inflammatory factors—sputum neutrophils and nitrogen oxides [16]. Therefore, ectoine inhalation solution can be expected to be effective for the treatment of acute bronchitis and acute respiratory inflammations.

This study investigated ectoine inhalation solution for the treatment of acute bronchitis and/or acute respiratory infections. Its effectiveness, tolerability, and safety were compared with those of normal saline inhalation solution.

## 2. Methods

### 2.1. Study Design

This prospective, patient-preference, observational study involved nine study centers in Germany from December 2015 to April 2016 (Clinicaltrials.gov identifier: NCT 02632851). It was approved by the local ethics committee at the University Hospital Cologne (reference number: EK-2015-373) and conducted in accordance with the German Medical Devices Act (Medizinproduktegesetz, MPG), section 23b. The observational study design allows for investigation of the study medication under routine medical practice; thus, randomization and placebo control are not permitted. The decision to use ectoine inhalation solution or saline inhalation solution was made by the patients before being included. Informed consent to data use was provided by all patients.

We aimed to recruit 120 patients older than 5 years of age who suffered from acute bronchitis and/or acute respiratory infections. The study lasted 17 days, comprising an inclusion visit (V1, day 1), a final visit (V2, day 7), and a follow-up questionnaire (FU, day 17). Treatment lasted 7 days, being administered from V1 to V2.

### 2.2. Study Medications

The ectoine inhalation solution (Ectoin® inhalation solution, bitop AG, Dortmund, Germany) used in this study contained ectoine (1.3%) and sea salt in water. The saline inhalation solution (PARI NaCl inhalation solution, PARI GmbH, Starnberg, Germany) contained 0.9% NaCl in water. Patients applied the inhalation solution two to four times a day, with each application of 2.5 ml nebulized solution being dispensed using the inhalation device CompAIR™ (OMRON Medizintechnik Handelsgesellschaft mbH, Mannheim, Germany).

### 2.3. Effectiveness Variables

The general health of patients was assessed by the investigators at V1 and V2 on a 4-point scale (0 = very poor, 1 = poor, 2 = moderate, and 3 = good). Patients rated their general health in patient diaries and FU questionnaires on a scale from 0% (very poor) to 100% (very good).

The Bronchitis Severity Score (BSS) was used to assess the severity of five major bronchitis symptoms: cough, chest pain when coughing, dyspnea, expectoration, and auscultation findings on a 5-point scale (0 = no symptoms, 1 = mild, 2 = moderate, 3 = severe, and 4 = very severe). Symptoms were assessed by the investigators at V1 and V2, and by patients in the patient diaries and the FU questionnaire.

The general effectiveness of the treatment was evaluated by investigators and patients independently at V2 using the Integrative Medicine Outcome Scale as “complete recovery”, “major improvement”, “slight/moderate improvement”, “no change”, or “deterioration”.

Sputum samples were analyzed using the Human XL Cytokine Array (R&D Systems, Minneapolis, MN, USA). Sputum samples were collected at V1 and V2 as described previously [16].

### 2.4. Tolerability and Safety Variables

The tolerability of the treatment was assessed by investigators and patients independently at V2 as “poor”, “satisfactory”, “good”, or “very good”. All adverse events were documented, and their relations to the treatment were evaluated by the investigators.

### 2.5. Statistical Analyses

Data were analyzed using the statistical software package SPSS version 23 (IBM Corp, Armonk, NY, USA). Data were entered in the database by two persons independently to avoid errors. The corrected database underwent a plausibility test for the range of single variables. The level of statistical significance was set to 5% for all tests. The BSS and individual symptoms scores were analyzed descriptively and tested for normal distribution using the Kolmogorov–Smirnov test. The Wilcoxon signed-ranks test was used to detect significant differences between baseline and final symptom scores. The variables of the general effectiveness, general tolerability of the treatment, and sputum samples were analyzed descriptively. Comparisons of the baseline-adjusted symptom scores between the two groups were analyzed using the Mann–Whitney* U* test. All statistical analyses were explorative. Figures were created using GraphPad Prism version 8.0 (GraphPad Software Inc., La Jolla, CA, USA).

## 3. Results

### 3.1. Study Population

In all, 135 patients were recruited; 79 patients (58.5%) received ectoine inhalation solution and 56 (41.5%) saline inhalation solution. Patients were 5 to 87 years of age; the mean age was similar between the ectoine (45.0 years) and the saline groups (45.3 years). Most patients were adults (ectoine: 91.1%, saline: 94.6%). There were more female than male patients in both groups (ectoine: 63.3% female, 36.7% male; saline: 63.6% female, 36.4% male); the distribution of female and male patients was similar between both groups. About 50% of patients suffered from bronchitis and 50% from bronchitis and acute respiratory infections (ectoine group: 46.8% and 48.2% of patients; saline group: 48.2% and 48.2% of patients, respectively); the diagnoses of two patients in each group were not specified. There were more patients who smoked >10 cigarettes/day in the ectoine group (7.6%) than in the saline group (3.6%). There were also more asthma patients in the ectoine group (6.3%) than in the saline group (1.8%) (Table 1).

One patient in each group did not appear at V2. Six patient diaries and 13 FU questionnaires were not submitted. Data of these patients were included in the analysis using the last-value-carried-forward method.

### 3.2. Improvement in Bronchitis Symptoms

**Figure 1:**
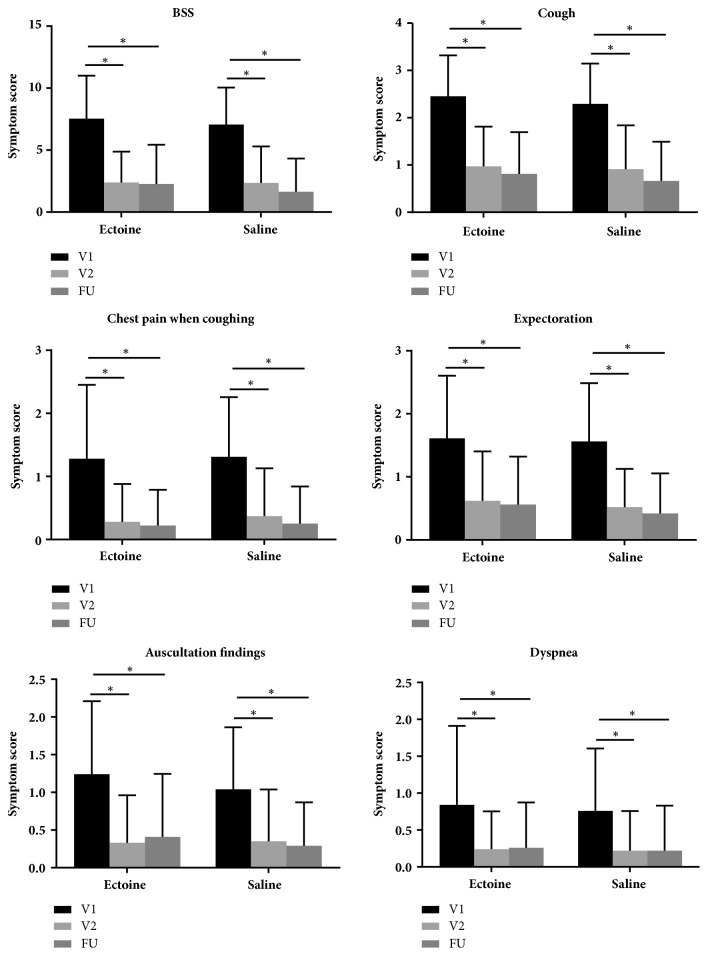
Symptom scores (mean ± SD; *∗*P < 0.05). BSS: Bronchitis Severity Score; FU: follow-up; V1: Visit 1; V2: Visit 2.

The physicians' assessments showed that the BSS and the individual scores for the symptoms of cough, chest pain when coughing, auscultation findings, dyspnea, and expectoration decreased significantly in both groups from V1 to V2 (*P* < 0.05) (Figure 1, Table S1). The baseline-adjusted scores were slightly greater in the ectoine group than in the saline group; the differences in these scores between the two groups were not significant (*P >* 0.05; Table 2). From V2 to FU, symptoms improved slightly in both groups (Figure 1, Table 2).

**Figure 2:**
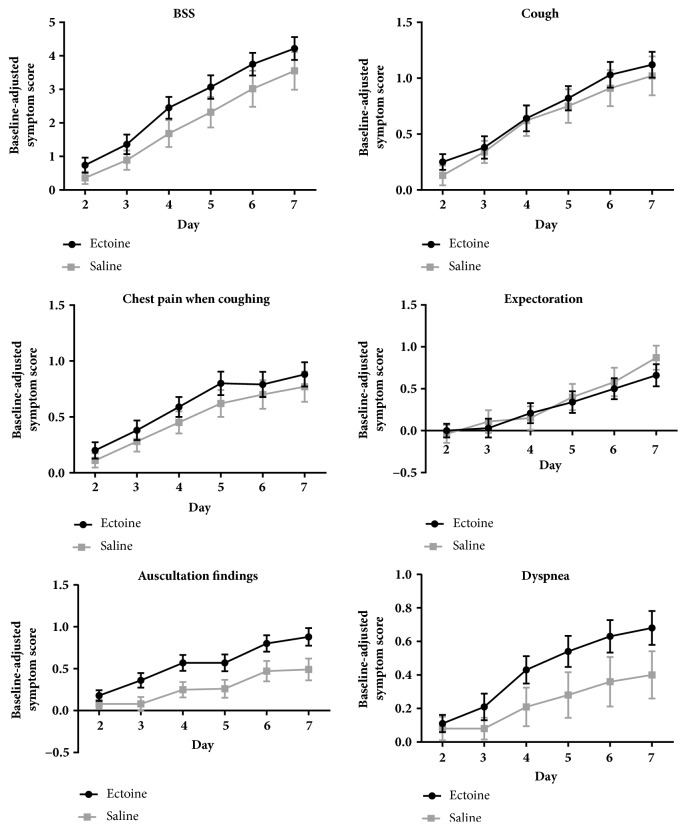
Baseline-adjusted symptom scores (mean ± SEM; analyses of the area under the curve are presented in Table 3). BSS: Bronchitis Severity Score; SEM: standard error of the mean.

The patients' assessments also indicate that symptoms improved more in the ectoine group than in the saline group. However, comparisons of the baseline-adjusted scores did not yield significant differences between the two groups (*P *> 0.05; Figure 2, Table 3). The differences in the area under the curve for the symptoms of dyspnea (*P =* 0.031) and auscultation findings (*P =* 0.011) were significant in favor of ectoine (Table 3).

Symptoms of patients treated with ectoine improved earlier than those of patients treated with saline. The first significant reduction in the score for the symptoms of cough, chest pain when coughing, dyspnea, and auscultation findings was reported beginning on day 2 in the ectoine group (*P* < 0.05) and beginning on day 3 (cough), day 3 (chest pain when coughing), day 5 (dyspnea), and day 4 (auscultation findings) in the saline group (*P* < 0.05). The first significant reduction in the score for the symptom of expectoration began on day 5 in both groups (*P* < 0.05, Table S1).

### 3.3. General Effectiveness of the Treatment

After treatment, more patients in the ectoine group (73.0%) than in the saline group (67.3%) assessed their condition as “completely recovered” or “greatly improved”. Similarly, more physicians (76.6%) assessed patients treated with ectoine as being “completely recovered” or “greatly improved” than did so (72.2%) for patients treated with saline.

### 3.4. Improvement in General Health

The physicians' assessments showed that the patients' general health improved in both groups from V1 to V2. The mean general health score increased by 40.7% in the ectoine group and by 35.8% in the saline group from V1 to V2. In analogy to the physicians' assessments, the patients' assessments also showed that general health improved in both groups. From V1 to V2, the general health score increased by 30.6% in the ectoine group and by 30.6% in the saline group. From V1 to FU, it increased by 79.3% in the ectoine group and by 80.2% in the saline group. The first significant increase in the general health scores was reported beginning on day 2 in both groups.

### 3.5. Tolerability and Safety

Almost all patients and physicians assessed the ectoine inhalation solution (96.1% of patients and 97.4% of physicians) and the saline inhalation solution (96.4% of patients and 96.4% of physicians) as being “good” or “very good”.

Two treatment-related adverse events occurred in the ectoine group and one in the saline group. In the ectoine group, one patient had nausea and headache immediately after inhalation, and the other patient had a dry cough that interrupted inhalation. In the saline group, one patient had a burning sensation in the throat 20 minutes after inhalation. No interventions were needed for these events. No fatalities or serious adverse events occurred in this study.

### 3.6. Sputum Analyses

Sputum samples were obtained from 11 patients. Samples from four patients receiving ectoine and two receiving saline were adequate for the assay. Our analysis showed that the changes from V1 to V2 in the levels of three growth factors—angiopoietin-1, epidermal growth factor, and interleukin-10—in the ectoine group were greater than those in the saline group (data not shown).

## 4. Discussion

The results of this study suggested that ectoine inhalation solution seems to be slightly more effective than normal saline inhalation solution in the treatment of acute bronchitis and acute respiratory infections. The first significant reductions in symptom scores occurred earlier in the ectoine group than in the saline group. Patients and physicians rated the effectiveness of ectoine slightly more favorably than that of saline inhalation solution. Symptom score reductions were slightly greater in the ectoine group than in the saline group. The analysis of the area under the curve for the symptoms of dyspnea and auscultation findings was significantly greater in the ectoine than in the saline group, albeit in the range expected when testing more than 20 independent variables.

Because this study did not include a placebo control, we compared the BSS reductions observed in this study with those from previous studies. Similar to our study, those studies were also conducted using natural, nonpharmacological treatment modalities for bronchitis. In two studies investigating an herbal preparation from* Pelargonium sidoides, *one study showed that the mean BSS decreased by 4.3 ± 1.9 to 6.3 ± 2.0 (depending on the dose) in the active treatment group and by 2.7 ± 2.3 in the placebo group. The other study showed that the mean BSS decreased by 7.2 ± 3.1 in the active treatment group and by 4.9 ± 2.7 in the placebo group [17, 18]. In another study, the combination of thyme herb with primrose root reduced the mean BSS by 6.2, and the placebo reduced the same mean by 4.1 [19]. In yet another study, the combination of thyme herb with ivy leaves reduced the mean BSS by 6.6, whereas placebo reduced it by 5.0 [20]. Thus, the reduction in the mean BSS of 5.13 ± 3.11 in the ectoine group in our study is greater than the reductions reported for the abovementioned placebo groups. However, the reduction is smaller than that of the placebo group in another study using* Pelargonium sidoides *[21]. Ectoine seems to be inferior to* Pelargonium sidoides*, the combination of thyme herb and primrose root, and the combination of thyme herb with ivy leaves for the treatment of bronchitis. However, it is important to note that the herbal drug* Pelargonium sidoides* may cause hepatotoxicity [22]. Nevertheless, because of varying baseline parameters, direct comparisons of the BSS between studies are informative only to a limited extent.

This observational study was designed in accordance with the German Medical Devices Act, section 23b, and aimed to investigate the study medications under routine clinical practice. A placebo control is not allowed in this study design; instead, we compared ectoine inhalation solution with an active control—normal saline inhalation solution. Although this study was nonrandomized, the similar demographic characteristics at baseline allowed for a comparison of the effectiveness between the two treatments.

## 5. Conclusions

In this pilot study, conducted under real-life conditions, ectoine inhalation solution seems to be slightly more effective than saline inhalation solution for the treatment of acute bronchitis and acute respiratory infections. It acts faster than saline inhalation solution does. Furthermore, patients and physicians assessed ectoine inhalation solution slightly more favorably than saline inhalation solution.

## Figures and Tables

**Table 1:** Patient characteristics at baseline.

**Treatment**		**Ectoine**	**Saline**
**Number of patients (**%**)**		79 (58.5%)	56 (41.5%)

**Age (years)**	Mean ± SD	45.0 ± 20.0	45.3 ± 18.8
	Median	49.0	47.5
	Minimum	5	5
	Maximum	87	83

**Age group N (**%**)**	5–11 years old	3 (3.8%)	2 (3.6%)
	12–17 years old	4 (5.1%)	1 (1.8%)
	≥18 years old	72 (91.1%)	53 (94.6%)

**Sex N (**%**)**	Female	50 (63.3%)	35 (63.6%)
	Male	29 (36.7%)	20 (36.4%)

**Diagnosis N (**%**)**	Acute bronchitis	37 (46.8%)	27 (48.2%)
	Acute bronchitis and acute respiratory infections	40 (48.2%)	27 (48.2%)

**Patients who smoked >10 cigarettes/day (N (**%**))**		6 (7.6%)	2 (3.6%)

**Asthma patients (N (**%**))**		5 (6.3%)	1 (1.8%)

N: number; SD: standard deviation.

**Table 2:** Reductions in symptom scores from V1 to V2 and from V1 to FU.

**Variables**	**Treatment**	**Reduction in symptom score from V1 to V2 (mean ± SD)**	**Reduction in symptom score from V1 to FU (mean ± SD)**
**BSS**	Ectoine	5.13 ± 3.11	5.35 ± 3.75
	Saline	4.71 ± 3.57	5.40 ± 2.98

**Cough**	Ectoine	1.48 ± 1.06	1.66 ± 1.09
	Saline	1.38 ± 1.25	1.60 ± 1.10

**Chest pain when coughing**	Ectoine	1.00 ± 1.05	1.06 ± 1.16
	Saline	0.94 ± 0.92	1.08 ± 0.81

**Auscultation findings**	Ectoine	0.91 ± 0.96	0.83 ± 1.16
	Saline	0.69 ± 0.88	0.76 ± 0.78

**Dyspnea**	Ectoine	0.61 ± 0.94	0.57 ± 1.08
	Saline	0.54 ± 0.91	0.53 ± 0.90

**Expectoration**	Ectoine	0.99 ± 0.97	1.13 ± 1.08
	Saline	1.04 ± 1.01	1.15 ± 0.98

BSS: Bronchitis Severity Score; FU: follow-up; SD: standard deviation; V1: Visit 1; V2: Visit 2.

**Table 3:** The area under the curve of baseline-adjusted symptom scores shown in Figure 2.

** Variables**	**Ectoine** **(mean ± SD)**	**Saline** **(mean ± SD)**
**BSS**	15.58 ± 13.88	11.81 ± 15.73

**Cough**	4.24 ± 4.52	3.77 ± 5.20

**Chest pain when coughing**	3.64 ± 4.42	2.94 ± 4.07

**Dyspnea** **∗**	2.61 ± 3.71	1.40 ± 4.29

**Expectoration**	1.74 ± 5.39	2.08 ± 5.59

**Auscultation findings** **∗**	3.36 ± 4.29	1.62 ± 3.70

*∗P* < 0.05 between groups.

BSS: Bronchitis Severity Score; SD: standard deviation.

## Data Availability

The data used to support the findings of this study are available from the corresponding author upon request.

## Abbreviations

BSS: Bronchitis Severity Score
FU: Follow-up
SD: Standard deviation
V: Visit.

## Supplementary Materials

Supplementary Materials
** Supplementary Table** **1.** Exact* P* values for the analyses of (1) baseline adjusted symptom scores (V2 – V1) as assessed by the investigators, (2) AUCs, and (3) daily baseline adjusted symptom scores as assessed by the patients.
