# Supplementary material for: Ectoine-Containing Inhalation Solution versus Saline Inhalation Solution in the Treatment of Acute Bronchitis and Acute Respiratory Infections: A Prospective, Controlled, Observational Study
Source: Biomed Res Int. 2019 Jan 31;2019:7945091. doi: 10.1155/2019/7945091 (PMC6374829; doi:10.1155/2019/7945091)
Supplement: Supplementary Materials — Supplementary Table 1. Exact P values for the analyses of (1) baseline adjusted symptom scores (V2 – V1) as assessed by the investigators, (2) AUCs, and (3) daily baseline adjusted symptom scores as assessed by the patients. [file 7945091.f1.docx]

**P values for the analyses of baseline symptom scores (V2 – V1) in each treatment group (as assessed by the investigators)**

| Treatment | Patients’ general health | BSS | Cough | Chest pain when coughing | Expectoration | Auscultation findings | Dypsnea |
| --- | --- | --- | --- | --- | --- | --- | --- |
| Ectoine | < 0.0001 | < 0.0001 | < 0.0001 | < 0.0001 | < 0.0001 | < 0.0001 | < 0.0001 |
| Saline | < 0.0001 | < 0.0001 | < 0.0001 | < 0.0001 | < 0.0001 | < 0.0001 | < 0.0001 |

**P values for the analyses of AUCs comparing the two treatment groups (data from the patient diaries)**

|  | BSS | Cough | Chest pain when coughing | Dyspnea | Expectoration | Auscultation findings |
| --- | --- | --- | --- | --- | --- | --- |
| *P* | .317 | .893 | .604 | **.031** | .848 | **.011** |

**P values for the analyses of baseline adjusted symptom scores as assessed by the patients**

| **BSS** | | | | | | | | | | | | | | | |
| --- | --- | --- | --- | --- | --- | --- | --- | --- | --- | --- | --- | --- | --- | --- | --- |
| Treatment | Day 2 –Day 1 | Day 3 – Day 1 | Day 4 – Day 1 | | Day 5 – Day 1 | | | Day 6 – Day 1 | | Day 7 – Day 1 | | Day 8 – Day 1 | | Day 9 – Day 1 | Day 10 – Day 1 |
| Ectoine | .001 | < 0.0001 | < 0.0001 | | < 0.0001 | | | < 0.0001 | | < 0.0001 | | < 0.0001 | | < 0.0001 | < 0.0001 |
| Saline | .041 | .003 | < 0.0001 | | < 0.0001 | | | < 0.0001 | | < 0.0001 | | < 0.0001 | | < 0.0001 | < 0.0001 |
| **Cough** | | | | | | | | | | | | | | | |
| Treatment | Day 2 – Day 1 | Day 3 – Day 1 | Day 4 – Day 1 | | Day 5 – Day 1 | | | Day 6 – Day 1 | | Day 7 – Day 1 | | Day 8 – Day 1 | | Day 9 – Day 1 | Day 10 – Day 1 |
| Ectoine | .001 | < 0.0001 | < 0.0001 | | < 0.0001 | | | < 0.0001 | | < 0.0001 | | < 0.0001 | | < 0.0001 | < 0.0001 |
| Saline | .149 | .002 | < 0.0001 | | < 0.0001 | | | < 0.0001 | | < 0.0001 | | < 0.0001 | | < 0.0001 | < 0.0001 |
| **Chest pain when coughing** | | | | | | | | | | | | | | | |
| Treatment | Day 2 – Day 1 | Day 3 – Day 1 | Day 4 – Day 1 | | Day 5 – Day 1 | | Day 6 – Day 1 | | Day 7 – Day 1 | | Day 8 – Day 1 | | Day 9 – Day 1 | | Day 10 – Day 1 |
| Ectoine | .010 | < 0.0001 | < 0.0001 | | < 0.0001 | | < 0.0001 | | < 0.0001 | | < 0.0001 | | < 0.0001 | | < 0.0001 |
| Saline | .083 | .005 | < 0.0001 | | < 0.0001 | | < 0.0001 | | < 0.0001 | | < 0.0001 | | < 0.0001 | | < 0.0001 |
| **Dypsnea** | | | | | | | | | | | | | | | |
| Treatment | Day 2 – Day 1 | Day 3 – Day 1 | Day 4 – Day 1 | Day 5 – Day 1 | | | Day 6 – Day 1 | | Day 7 – Day 1 | | Day 8 – Day 1 | | Day 9 – Day 1 | | Day 10 – Day 1 |
| Ectoine | .046 | .012 | < 0.0001 | < 0.0001 | | | < 0.0001 | | < 0.0001 | | < 0.0001 | | < 0.0001 | | < 0.0001 |
| Saline | .317 | .248 | .098 | .039 | | | .016 | | .009 | | .013 | | .009 | | .009 |
| **Expectoration** | | | | | | | | | | | | | | | |
| Treatment | Day 2 – Day 1 | Day 3 – Day 1 | Day 4 – Day 1 | | Day 5 – Day 1 | | | Day 6 – Day 1 | | Day 7 – Day 1 | | Day 8 – Day 1 | | Day 9 – Day 1 | Day 10 – Day 1 |
| Ectoine | .991 | .821 | .111 | | .011 | | | < 0.0001 | | < 0.0001 | | < 0.0001 | | < 0.0001 | < 0.0001 |
| Saline | .634 | .440 | .327 | | .021 | | | .003 | | < 0.0001 | | < 0.0001 | | < 0.0001 | < 0.0001 |
| **Auscultation findings** | | | | | | | | | | | | | | | |
| Treatment | Day 2 – Day 1 | Day 3 – Day 1 | Day 4 – Day 1 | | | Day 5 – Day 1 | | Day 6 – Day 1 | | Day 7 – Day 1 | | Day 8 – Day 1 | | Day 9 – Day 1 | Day 10 – Day 1 |
| Ectoine | .006 | < 0.0001 | < 0.0001 | | | < 0.0001 | | < 0.0001 | | < 0.0001 | | < 0.0001 | | < 0.0001 | < 0.0001 |
| Saline | .157 | .371 | .012 | | | .019 | | < 0.0001 | | .001 | | < 0.0001 | | < 0.0001 | .001 |
| **Patients’ general health** | | | | | | | | | | | | | | | |
| Treatment | Day 2 – Day 1 | Day 3 – Day 1 | Day 4 – Day 1 | | | Day 5 – Day 1 | | Day 6 – Day 1 | | Day 7 – Day 1 | | Day 8 – Day 1 | | Day 9 – Day 1 | Day 10 – Day 1 |
| Ectoine | < 0.0001 | < 0.0001 | < 0.0001 | | | < 0.0001 | | < 0.0001 | | < 0.0001 | | < 0.0001 | | < 0.0001 | < 0.0001 |
| Saline | .006 | < 0.0001 | < 0.0001 | | | < 0.0001 | | < 0.0001 | | < 0.0001 | | < 0.0001 | | < 0.0001 | < 0.0001 |
